# A Growl from an Ignorant Man against Some Scientific Technicalities

**Published:** 1867-05

**Authors:** T. D’or’Emieulx


### A GROWL FROM AN IGNORANT MAN AGAINST SOME SCIENTIFIC TECHNICALITIES.

Read before the American Microscopical Society of New York, March 23d, 1867.

BY T. D'OR'EMIEULX.

It is a fact much to be regretted, that, in almost every department of science, the same want of precision and explicitness should occasionally be met with in many of the technicalities made use of by scientific men. It is no less to be regretted that these same learned gentlemen should deem it both decorous and necessary, to overload their vocabulary with such an innumerable quantity of these same technicalities, when the plain mother tongue would answer just as well. Since the day when that incongruous apposition of words, "POLARIZATION OF LIGHT," was agreed upon to designate that brilliant phenomenon, so gorgeously displayed by the microscope, to our present day, there seems to have been wilful and premeditated endeavor to shut up science within the walls of an impregnable fastness, guarded by those untamed and untamable Greek monsters, ready to devour any timid intellect attempting to pass the threshold of the sacred inclosure.

Nor is our favorite pursuit free, by any means, from the aforesaid reproach. Indeed, I think it is, more than any other branch of learning, liable to the most serious charges in that respect. What, for instance, does the word "Infusoria" mean? Does it convey to the mind the remotest idea of what it is intended to represent? Is it sufficient to say that the name assigned originally to those minute organisms, which mysteriously enough spring up in any vegetable infusion, has been preserved and extended to other organisms by routine, though these latter may be as remote from the former as they possibly can be? Genera present here and there analogous difficulties. For instance, shall we call this valve a gyrosigma, or more simply a plurosigma, or again still more simply, a navicula? But when we consider species, the confusion becomes still greater.

The qualifying adjective changes according to every man's affections, preferences, self-conceit or convenience. The Latinized name of a learned Professor is affixed to a certain subject by one, discarded and frowned down by another, who replaces it by his own name, or by some descriptive Greek combination. In the meantime the outside student, striving, sweating, struggling and wading through this confusion, is left in an inextricable muddle, from which he emerges at last more befogged, and more ignorant than ever.

This is certainly an evil which requires correction at the hands of those, in whose power it is to give tone and direction to scientific pursuits. Is not science sufficiently arduous to acquire, without making it next to inaccessible to the outside student, by unnecessary, injudicious and especially undescriptive technicalities.

But returning to the microscope, let us take its optical nomenclature, and see how far we can boast of sufficient clearness and precision in the choice of terms. Are we very sure, for instance, that we all understand what is meant by these fractions of the inch, used to indicate the magnifying power of objectives! When we speak of a $\frac{1}{4}$ inch glass, do we intend any well understood degree of amplification? No indeed. Well then, the inference is that the expression implies the working distance from the bottom glass of the front combination of the objective, to the object under observation when perfectly defined. We are all aware that it does not. What does it mean then? It means what you all know, namely, that if a single lens were made the object glass of a compound microscope, (by the by they tried the name of Engiscope for the compound microscope; but it would not take,) and it were required to obtain the same amplification as that shown by a $\frac{1}{4}$ inch achromatic objective, it would be necessary that that single lens be of a $\frac{1}{4}$ inch focus, to produce the same power. Well, if this is not a far fetched, round-about-turn to mislead most effectually, the unsophisticated student, I do not know what is.

The amplifying power of a glass expressed in diameters, taking for a standard, the eye piece A, would have been too clear and too perspicuous, it would never have done, and accordingly, was discarded; and this is not all, not only does the present appellation mislead, at first, as to the working distance of a glass, but it conveys no distinct idea of its capacities.

It is a well known fact, that no two objectives of a given denomination have identically the same amplifying power, even from the same maker. The consequence is that the optician, in delivering an object glass, labeled on its box 1-5, is only bound to give you a good working glass, fulfilling certain conditions of penetration and definition, but without any reference to its amplifying qualities. On the contrary, were the objectives named from their amplifying power, expressed in diameters, every purchaser of any object glass would at once know what he has a right to expect.

Let us take another instance of unsettled confusion. Are all opticians, and all microscopists, well agreed on the real meaning of the words "penetrating power?" I doubt it. Does it mean as Dr. Goring has it? "The work performed by the objective, owing to its angular apperture, in other terms, owing to its capacity to admit the greatest possible amount of light, transmitted by the object under observation"? Or should it assume the meaning assigned to it by Carpenter, who eschewing entirely the idea of angular aperture, as connected with the penetration, defines the latter, "the power of an objective glass to show, with an almost equal degree of distinctness, that part of an object which from its arrangement on the slide, or from its difference of thickness in its various parts, is in those parts out of focus"? *Ad huc sub judice lis est.* The question is not altogether decided; in the meantime, we, outsiders, are waiting in the cold till the friends of science come to our rescue and settle, beyond question or cavil, which is which.

It is high time in these days of popularization of science, that all discrepancies, misnomers, incommensurable polysyllables to express the simplest every day things, should be done away with, wherever a plain English word is available, and certainly it should be so in those publications intended for the general reader. Of course, technicalities, incomprehensible to the uninitiated, cannot, in many cases, be dispensed with, but let them in all instances be, as far as practicable, confined to the text-books and to the lecture room. Let scientific men not write, especially in reviews and quarterlies, only for their craft; let them make themselves comprehensible to all, let them remember that there is a large number of unsophisticated readers, who thirst for knowledge, and who will bless their efforts if they will only condescend to speak in the simple, plain mother tongue, whichever it may be.

The days when science, in the laboratory of the alchemist of old, wrapped itself up in a black cossack, constellated with frightful scarlet hieroglyphics, to impose upon the "*profanum vulgus*" are gone by. It may be well for the chestnut to be wrapped up, in its thorny and prickly envelope, to protect its growing kernel from the thievish paw and ravenous tooth of the squirrel, and of all that tribe of "rodentia," (a pretty word, by the by, used instead of the very plain English expression "gnawing tribe.") But the kernel of science needs no such protection. Science must inhabit a palace of the purest crystal, with a thousand gates; it must be seen from every point, be accessible to all, from every side, it must radiate from within, in every direction. Let us have science made inviting, attractive and fascinating to all. Dressed as it is in its useless technicalities, its indefinite misnomers, its undescriptive appellations, it becomes simply, to say the least of it, absolutely discouraging for those inquisitive minds which deprived of an early scientific training, wish at some later period in life, to bestow their study on some of the wonders that it has pleased the Creator of the universe, to scatter so lavishly before our eyes.

